# Study protocol: biomechanical testing, finite element analysis and prospective, randomized, clinical study of single screw cephalomedullary nailing versus integrated dual interlocking screw fixation for unstable (31A2_1–3_) intertrochanteric fractures in patients > 70 years old

**DOI:** 10.1186/s13018-023-04009-8

**Published:** 2023-07-28

**Authors:** Andreas Panagopoulos, Evangelia Argyropoulou, Zinon T. Kokkalis, Nicolaos Parchas, Konstantinos Tserpes

**Affiliations:** 1 Department of Orthopaedics, Patras University Hospital, Medical School, Patras University, Papanikolaou 1, 26504 Rio-Patras, Greece; 2 Laboratory of Technology and Strength of Materials, Department of Mechanical Engineering and Aeronautics, University of Patras, Patras, Greece

**Keywords:** Intertrochanteric fractures, Biomechanical testing, Finite element analysis, Single cephalomedullary nailing, Integrated dual interlocking screw nailing

## Abstract

**Background:**

Hip fractures are an increasingly common consequence of falls in older people that are associated with a high risk of death and reduced function. The vast majority of intertrochanteric fractures require surgical treatment to withstand early mobilization and weight bearing, which prevents complications due to prolonged bed rest and aids in fracture healing.

**Methods:**

This study is compromised by two parts, the experimental study and the clinical part. In the first part, a standard 130° nail will be used with the appropriate lag screw(s) and dynamic distal locking in synthetic osteoporotic femurs and the transmission of forces in the proximal femur, measured by a cortical surface-strain distribution, will be evaluated using digital image correlation. Finite element parametric models of the bone, the nails and their interface will be also developed. Finite element computations of surface strains in implanted femurs, after being validated against biomechanical testing measurements, will be used to assist the comparison of the nails by deriving important data on the developed stress and strain fields, which cannot be measured through biomechanical testing. In the other part, will set up a prospective, randomized, comparative clinical study among the Gamma3 and IT cephalomedullary nailing, in order to investigate if there are any statistical important differences in the main radiological measurements and functional status in closed unstable intertrochanteric fractures (A21-3) in patients aged over 70 years old at the 24-week follow-up evaluation using patient reported disease-specific outcomes.

**Discussion:**

This study will be the first to compare clinical, radiological and biomechanical measurements of the two different cephalomedullary nails. Our main hypothesis is that the IT nail would provide better radiological outcome and probably better clinical results than the Gama3 nail.

*Clinical trial registration* International Standard Randomized Controlled Trial Number (ISRCTN): https://doi.org/10.1186/ISRCTN15588442, registered on 19/4/2022.

## Introduction

Hip fractures are an increasingly common consequence of falls in older people that are associated with a high risk of death and reduced function [1, 2]. In fact, hip fractures constitute one of the most common impairments worldwide in terms of loss in disability-adjusted years for people older than 60 years old [1, 3]. The absolute number of hip fracture hospitalizations in the US are estimated to approach 289,000 in 2030 with an expected increase to 4.5 million by the year 2050 [4, 5]. In the UK, there is an ongoing age-standardized fall of hip fractures of 0.5% per year, but it is estimated that the annual incidence will double in the next 25 years [6]. The consequences for hip fractures in elderly individuals are significant in terms of lives lost and the associated negative impacts on hip fracture patients’ functioning and quality of life [7, 8]. In a recent critical review of 38 studies by Dyer et al. [1], hip fracture survivors experienced significantly worse mobility, independence in function, health, quality of life and higher rates of institutionalization than age matched controls. Even with integrated, multidisciplinary models for the treatment of hip fragility fractures the in-hospital mortality rate was 2.4%, and the overall mortality at 1 year from the intervention 18.7%; full mobility status or a low impairment of mobility was reached 32.1% of patients [9]. The reported mortality rate at 1 year in a very recent study from the Swedish Hip Fracture Registry [10] was approximately 20% for women and 30% for men in a total population study of 289,603 first hip fractures during a 20-year-old period (1998–2017).

The vast majority of intertrochanteric fractures require surgical treatment to withstand early mobilization and weight bearing, which prevents complications due to prolonged bed rest and aids in fracture healing. The type of surgery is generally based on fracture pattern and patient characteristics and is usually carried out with Dynamic Hip Screw (DHS) devices or cephalomedullary nails (Proximal femoral nails [PFN], PFN-antirotation nails [PFNA], Gamma nails [GN], Trigen Intertan [IT] nails or other implants) [11–16]. The optimal device for surgical fixation of trochanteric fractures remains under debate [17–33]. Considering the substantial burdens attributed to the management of intertrochanteric fractures, we believe that more evidence is required to evaluate the effectiveness of a single lag cephalomedullary screw vs dual integrated screw nailing for unstable intertrochanteric fractures. The aims of the present study are:

- to perform a biomechanical testing between IT and Gamma3 in relation to axial stiffness, torsional stiffness, and failure load in unstable intertrochanteric fractures created in 4th-generation osteoporotic sawbones;
- to create finite element parametric models of the bone, the nails and their interface; finite element predictions of surface strains in implanted femurs, after being validated against biomechanical testing measurements, will be used to assist the comparison of the nails by deriving important data on the developed stress and strain fields, which cannot be measured through biomechanical testing. Such parameters including non-central lag screw(s) position in either the anteroposterior or lateral plane and varus or valgus reduction.
- To compare the clinical and radiological results of the two implants in a prospective, randomized clinical study in patients over 70 years old with unstable AO/OTA A2 fractures with a minimum follow-up of 6 months.

## Materials and methods

### Biomechanical study

#### Implants

The experimental work will be undertaken in the Laboratory of Technology and Strength of Materials at the Department of Mechanical Engineering and Aeronautics of Patras University. Two implant systems will be compared against biomechanical testing, the classic Gamma3 and the InterTan nail (IT).

The Gamma3®nail (Stryker, Mahwah, NJ, USA) that would be used in the current study has a length of 180 mm, 11 mm nail diameter and 130° neck-shaft angle and utilizes a single lag screw with a standard diameter of 10.5 mm. The Trigen Intertan (Smith + Nephew, Watford, UK) nail is a current generation IM device, featuring a dual lag screw configuration comprised of a 11.0 mm superior lag screw and a 7.0 mm smaller integrated screw that allows for linear compression of the fragments at the fracture site while providing high rotational stability [32, 33]. The proximal part of the nail has a trapezoid design that provides a pressfit fixation in the metaphyseal region thus positioning more material on the lateral side of the nail where tensile/stretching forces tend to be greatest. Finally, the “clothes pin” distal tip provides less rigidity to decrease the stress riser and reduce the incidence of anterior thigh pain. An 11 mm nail with a 130° neck-shaft angle will be used for the present study.

We would hypothesize that the above variations, may create differences in strain distribution and thus dissimilar biomechanical behaviors of these two nails. The main hypothesis of our biomechanical study is that the InterTan nail would provide higher stiffness and load to failure and less varus collapse in unstable intertrochanteric fractures (AO/OTA A2). The transmission of forces as measured by cortical surface-strain distribution in the proximal femur will be evaluated using digital image correlation (DIC) and classic strain gauges.

#### Preparation of specimens

Eight synthetic composite osteoporotic femurs of medium size (model: 3503; SawBones Worldwide, WA, USA) with 10 PCF low-density cancellous, thin-walled low-density cortical shell and 16 mm hollow canal, would be randomly assigned to two groups (*n* = 4 in each group) to receive either a classic Gamma3 or an IT nail. Two additional specimens will be kept intact and will be served as reference for system calibration and material properties validation. The use of sawbone models with validated mechanical properties [34–36] was preferred instead of cadaveric bones to eliminate size, geometry and mechanical variability between specimens thus increasing stanchness of our numerical analysis due to the availability of the finite element 3D digital file with coded material properties of our specimen (Femur, Finite Element Model of #3503, SawBones Worldwide, WA, USA).

**Fig. 1:**
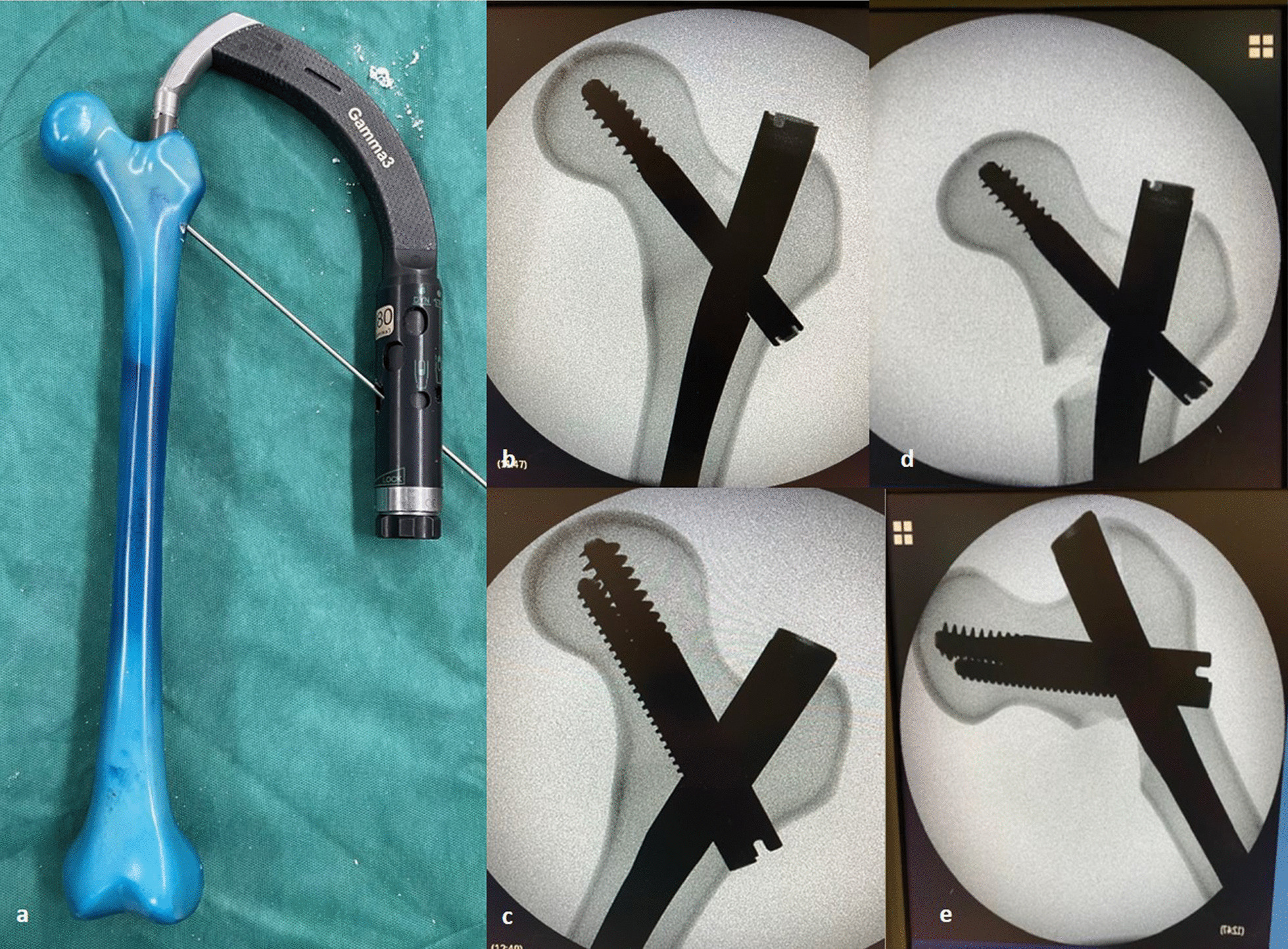
**a** The instrumentation of Gamma3 has been applied in a standard manner, according to the manufacturer’s manual in an osteoporotic Sawbone model, first on the intact sawbones; **b**–**d** Fluoroscopic images of Gamma3 in the intact and fractured Sawbone; **c**–**e** Fluoroscopic images of InterTan in the intact and fractured Sawbone

The instrumentation of Gamma3 and IT will be applied in a standard manner, according to the manufacturer’s manual, first on the intact sawbones (prior to fracture creation) under image intensifier using antero-posterior and lateral imaging to ensure proper implant position and a tip-to-apex distance (TAD) < 20 mm (Fig. 1a–c).

The instrumentation will be removed and the fractures would be created using an electric saw and a custom-made cutting guide. For the creation of an unstable intertrochanteric fracture (AO/OTA 31A2.2) the fracture line will be at an angle of 47° to the horizontal level running from the lesser trochanter followed by removal of an additional wedge of bone, containing the lesser trochanter. The sawbones will be re-instrumented and prepared for biomechanical testing (Fig. 1d, e).

**Fig. 2:**
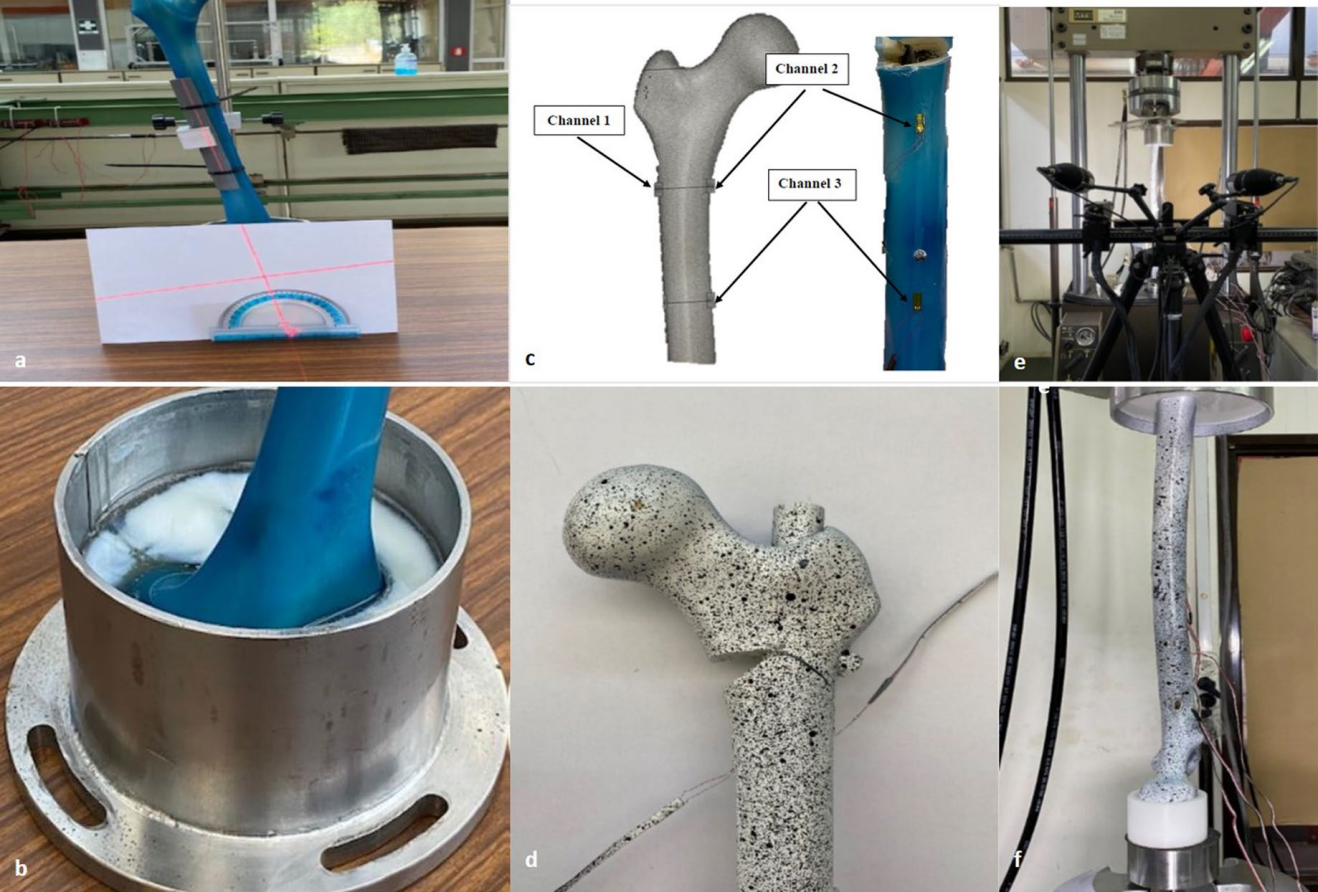
**a** position of the composite femur at 11° of abduction in the frontal plane and neutral on the sagittal plane; **b** Fixation of the specimen distally at the supracondylar level to a custom resin mold (Smooth-Cast 300 Series); **c** Standard strain gauges will be applied at the medial and lateral side of the specimen 2 cm below the fracture line as well as 2 cm below the distal interlocking screw; **d** A thin layer of white paint with black speckles will be applied at the proximal end of the femur to measure the transmission of forces, in the cortical surface-strain distribution using DIC; **e** and **f** the biomechanical testing machine and the mode of cycling loading

The natural inclination of the femur at the single-leg stance, which is 11° of abduction in the frontal plane and neutral on the sagittal plane will be ensured using the surface of the posterior condyle as reference point [37] (Fig. 2a). The femurs will then be fixed distally at the supracondylar level to a custom resin mold (Smooth-Cast 300 Series) properly reinforced with an orthogonal steel plate (Fig. 2b). Standard strain gauges (model C2A-06-125LW-350, 350Ω ± 0.6%, − 50° up to + 80 °C) will be applied at the medial and lateral side of the specimen 2 cm below the fracture line as well as 2 cm below the distal interlocking screw (Fig. 2c). Finally, a thin layer of white paint with black speckles (Fig. 2d) will be applied at the proximal end of the femur to measure the transmission of forces, in the cortical surface-strain distribution using digital image correlation (DIC) (Fig. 2e).

#### Loading scheme

Once the implants were settled well and distal ends were embedded, the two groups of model bones were fixed on the biomechanical test machine (MTS 647, hydraulic wedge grip) (Fig. 2f). Initially, all specimens will be subjected to an axial pressure of 100–200 N for 10 cycles with a loading rate of 1 Hz to eliminate creeping effect.

Axial cycling load compression will be applied to simulate the stress experienced by a patient with 70 kg body weight at 4–6 weeks postoperatively [38, 39]. Firstly, the axial compression test is carried out under cyclic loading, with an initial load of 400 N, incremental load of 100 N, and maximum load set to 1400 N, divided into 10 subgroups; each subgroup will be conducted in a cycle of 10,000 times, respectively, with a loading rate of 1 Hz. After the cyclic test, the average fracture gap movements will be recorded. A torsional test will follow with the following parameters: Starting from 0°, the maximum torsion angle will be set to 3° with a loading rate of 0.1°/s. Torque at the angle of 1°, 2°, 3° will be recorded, respectively. Finally, an axial compression failure test will be performed with a loading rate of 4.6 mm/s continuously, until fatigue failure; the latter is defined as fracture gap > 20 mm, nail cutting‐out or breakage and fracture line found near the distal locking screw.

#### Statistical analysis

The SPSS 19.0 statistical software package will produce the statistical analysis of the study. With the aid of Shapiro–Wilk test, the normal deviation of cycles to failure, failure load and axial construct stiffness in each group will be monitored. To screen the fluctuation in the parameters of interest, the Levene test will be utilized. Any major difference between the two implants will be registered with paired-samples *t* test, while we set a *p* = 0.05 as level of significance.

### Finite element analysis (FEA)

Finite element analysis of the implanted sawbones will be performed using the ANSYS v.19.2 commercial FE code (ANSYS inc. Cannonsburg, Pennsylvania, USA). The femoral geometry model will be obtained by the manufacturer (3D CAD digital file with coded material properties, Sawbones, Vashon Island, Washington, USA). The geometrical features of each implant (Gamma3 and IT) will be characterized following 3D CT scanning. Only the basic geometrical features will be modeled using FEA, since it has been recognized that several additional details (i.e., threads of the lag screw(s) or distal interlocking screw) significantly complicate the analysis, without affecting considerably the computed stress and strain fields [40].

**Fig. 3:**
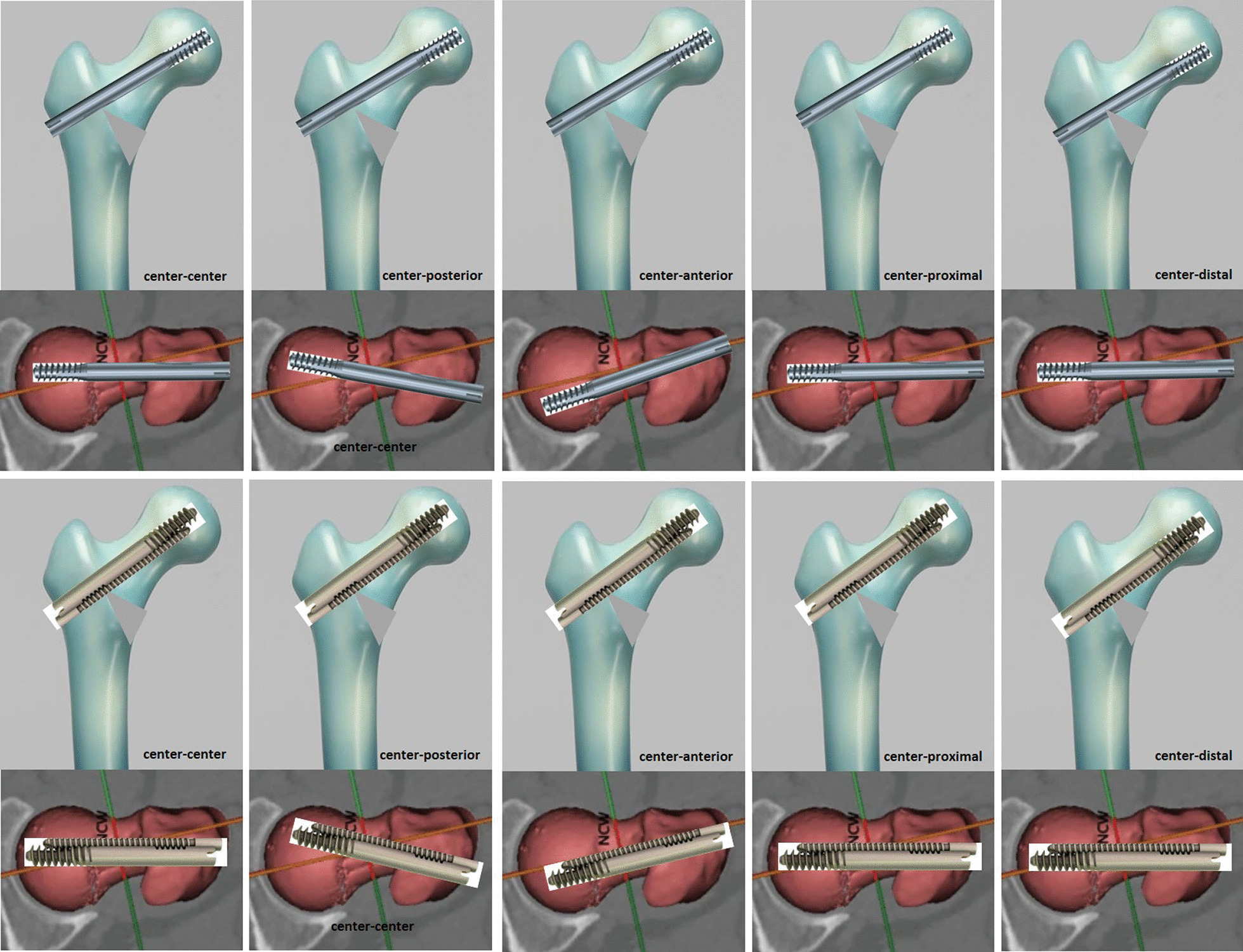
Several FEA models will be created with the aim to study different scenarios of reduction (in varus or valgus) as well as inappropriate lag screw positions

Sawbones geometry will be imported in the ANSYS FE code and the fracture plane will be modelled by creating a proper surface that divide the cortical and cancellous volumes of the bone for the unstable fracture cases. The native neck shaft angle and the anatomic reduction angle will be 135°. A central position of the hip screw with a TAD of 16–18 mm and a minimum of 2 mm width of cancellous bone between the hip screw and the femoral cortex will be applied. Several FEA models will be created with the aim to study different scenarios of reduction (in varus or valgus) as well as inappropriate lag screw positions as illustrated in Fig. 3. Our hypothesis is that even with inappropriate reduction or lag screw(s) mispositioning the biomechanical properties of IT will be better than Gamma3.

As has been proposed in a similar study [40], the developed geometries will be meshed using tetrahedral elements with an optimal element size of 3 mm as this leads to acceptable results regarding accuracy, without significantly increasing the computational cost. Appropriate linear elastic, isotropic and homogeneous material properties will be assigned to the several parts of the FE model, based on the data provided by the manufacturers. The longitudinal Young’s modulus for the cortical bone (82 pcf fiber filled epoxy) is E_cortical_ = 5.2 GPa, and for the cancellous bone (10 pcf solid foam) E_cancellous_ = 58 MPa, while for the Gamma3 and IT the titanium alloy the Young’s modulus is E_material_ = 116 GPa. For the modeling of the distal femoral fixation during the experiments, fully fixed boundary conditions will be applied on the FE models surface nodes situated at the lower 130 mm of the femur model. A constant vertical force of 1000 N will be applied on the top of the femoral head; as a linear-elastic FE analysis will be considered, the calculated results will be derived for other load values using linear interpolation or extrapolation. Linear regression modeling will be utilized in order to associate a set of independent variables or predictors (implant type, lag screw(s) positioning and reduction angle) with any measurement outcome, also referred to as the dependent variable or target, separately. For each model, the beta estimates and the *p* values will be returned, among others. Statistical significance was taken when *p* < 0.05.

### Clinical study

#### Design

This study will be a prospective, randomized clinical trial to compare the clinical and radiological outcome between single screw cephalomedullary nailing (Gamma3) and integrated dual interlocking nailing (InterTan-IT) for the treatment of unstable (31A2_1–3_) intertrochanteric fractures. The paper complies with the Standard Protocol Items: Recommendations for Interventional Trials (SPIRIT) 2013 Statement for reporting of clinical trial protocols. The study is sponsored by the University of Patras Research Committee (ELKE) (University of Patras Campus, Rio, Greece). Ethical approval has been already acquired by the Ethics Committee of the University Hospital of Patras (approval number: 3373/7-2-2022) and the patients’ written consent will be obtained before participating in the study. The study is listed with the International Standard Randomized Controlled Trial Number (ISRCTN): https://doi.org/10.1186/ISRCTN15588442 (registered on 19/4/2022).

#### Primary objective

The *primary aim* of this study is to investigate if there are any significant differences in the main radiological measurements (cut-out, varus displacement, loss of reduction, malunion, nonunion) between Gamma3 and IT in closed unstable intertrochanteric fractures (31A2_1–3_) in patients aged over 70 years old at the 24-week follow-up evaluation.

The *second primary aim* would be the functional status at the 24-week follow-up using patient reported disease-specific scores (Harris Hip score HHS-and Oxford hip score-OHS).

#### Secondary objectives (Table 1)

**Table 1:** Timeline chart of patient clinical evaluation

Outcome measure	Specification	Score	Assessment times				
			Pre-op	Post-op	6w	12w	24w
*Patient-reported outcome measures*							
EQ-5D-3L	General health status	5–15	√		√	√	√
SF-36	General health status	0–100	√		√	√	√
Elderly mobility scale	Fragility Assessment	0–20	√		√	√	√
SARC-F questionnaire	Sarcopenia assessment	0–10	√		√	√	√
NPRS	Joint pain	0–10	√	√	√	√	√
*Hip Joint-specific measures*							
Harris hip score (HHS)	Joint-specific score	0–100	√		√	√	√
Oxford hip score (OHS)	Joint-specific score	0–100	√		√	√	√
*Surgeon and patient related complications*			Intra-op	Post-op	√	√	√
Failure rates				Post-op	√	√	√

*NRPS* numerical pain rating scale, *SF-36* short-form 36, *EMS* elderly mobility scale, *SARC-F* strength, assistance with walking, rising from a chair, climbing stairs, and falls questionnaire, *HHS* Harris hip score, *OHS* Oxford hip score

Several secondary objectives will also be studied to evaluate the effectiveness and safety of the devices by quantifying and drawing presumptions from observed differences between treatment groups in the following:

- Comparison of perioperative and intraoperative surgical data (age-adjusted Carlson Comorbidity Index [41], operation time, fluoroscopy time and dosage, blood loss, length of hospital stay, prescription of pain-killers, osteoporosis assessment using postoperative DEXA of the unaffected hip, union time and intraoperative surgeon related complications, including lag screw malposition, propagation of the fracture, non-anatomical reduction, varus/valgus deformity, rotational deformity and tip-apex distance-TAD).
- Pain level at the perioperative period and at 6-, 12-, and 24-weeks postoperatively using the visual analogue scale (VAS) Pain Score.
- Patient reported general health status prior to surgery and at 6-, 12- and 24-weeks post-surgery using the SF-36 form, the EQ-5D-3L Questionnaire, the SARC-F Index and the Elderly Mobility Scale (EMS).

#### Patient selection

**Fig. 4:**
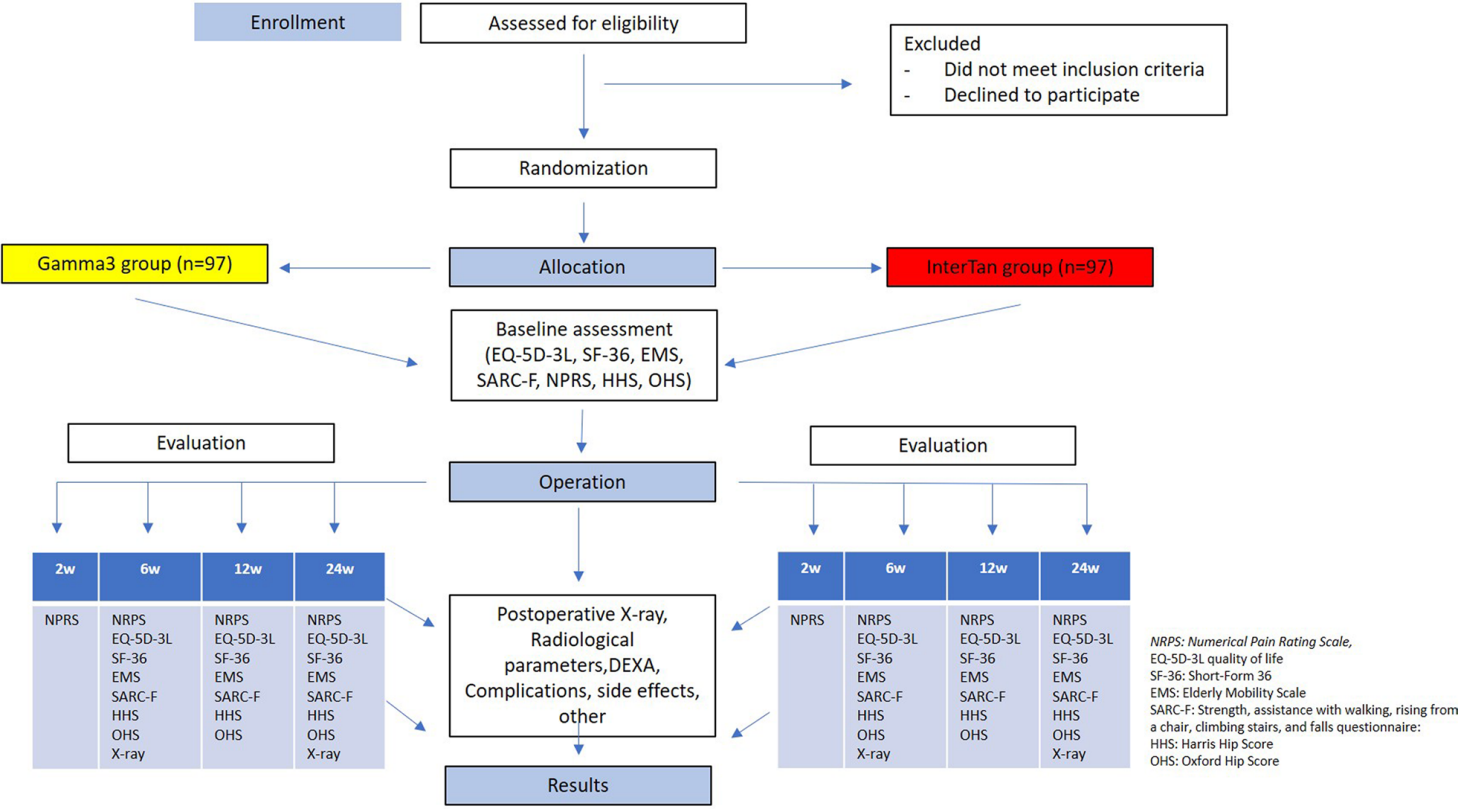
Flowchart of the clinical study

The patients that will take part in the study, are those over 70 years old, admitted in the Orthopaedic Clinic of the University Hospital of Patras, Greece, suffering from a closed unstable (31A2_1–3_) intertrochanteric fracture. Those patients will be randomized in two groups (Gamma3 and IT). The pre-operative data will be comprised of demographic information, radiological examination of the hips and other questionnaires to conclude the overall physical health and mobility prior the fracture. The participants will be free to withdraw from the study or the investigator can withdraw a patient. If a participant withdraws, they will be listed to a “Change of Status” form and will be invited to present for an endpoint follow-up (Fig. 4).

#### Eligibility criteria

1. Closed intertrochanteric fracture (AO/OTA:31A2).
2. Age over 70 years.
3. Presentation to hospital within 7 days of injury.
4. No concomitant injuries or prior operations to the unaffected hip.

#### Exclusion criteria

1. Concomitant injuries affecting treatment or rehabilitation of the affected limb.
2. Associated neurovascular injuries requiring immediate surgery.
3. Patients with limited Greek proficiency including family members.
4. Patients where consent is refused.
5. Patients with severe dementia, non-ambulated and with severe associated diseases prohibiting operative intervention.

#### Sample size

This study will use two primary clinical outcome measures namely the Oxford Hip Score and the Harris Hip Score. The minimum clinically important differences (MCIDs) for the HHS have been estimated between 7 and 10, whereas for the OHS between 5 and 7. The aim will be to recruit 78 patients in each group as this will provide sufficient participants to obtain a power of 90% for both primary clinical outcome measures. With an allowance for 15% drop-out, the total number of patients required will be 194 (97 in each group). If recruitment proves to be problematic during the course of the trial, then the target will be lowered and the more usual 80% power level will be considered sufficient. For this scenario, the total number of patients required will be 140 (including 15% for drop-out).

#### Randomization

Randomization sequence will be created using Stata 9.0 (StataCorp, College Station, TX) statistical software and will be stratified with a 1:1 allocation ratio using random block sizes of 2, 4, and 6. An investigator not involved in the main study will perform the randomization process. These data will be stored on computer and will be available at the day of the intervention.

#### Concealment mechanism

For each surgical session patient details and the randomized treatment will be sealed in an opaque envelope that will be given to the coordinated nurse just before the start of the intervention. The patients, relatives, investigators, nurses, and all relevant personnel would be blinded to the indented treatment. The envelope will be opened by the coordinated nurse after the patient is sedated and placed to the radiological table, the hip is properly prepared and the surgeons are ready to apply the intervention. Both instrumentations for Gamma3 or IT would be available at the operative room. After opening the envelope, the procedure will be unblinded for all participants.

### Surgical intervention

The patients for the study will be randomly divided in two groups. Group A: Gamma3 nail (Stryker) and Group B: InterTan (IT) nail (Smith & Nephew). The surgery will take place in one specific operating room with the standardized surgical technique, using the same c-arm for the intra-operative radiological evaluation. Anatomical reduction of the fracture will be intended in all cases prior to nail insertion. If this is not possible, several techniques of closed or open reduction will be applied in order to achieve an acceptable fixation of the fracture. The patients’ position will be on the traction table, using a minimal approach. The lag screw(s) in both groups will be put in the center of the femoral head in the lateral and anteroposterior X-ray view, while the Tip to Apex Distance (TAD) will be approximately 20 mm. All participants in both groups will receive the same physical therapy and adequate anti-osteoporotic treatment.

#### Radiographic evaluation

A standard technique will be kept, in order to have the same parameters in all radiographs. For the antero-posterior ones of the pelvis, both legs of the patients will be rotated inwards 15°. The radiographic measurements will be monitored by two distinguished examiners through RadiAnt DICOM Viewer. Intra-class correlation coefficients (ICC) will check the inter- and intra-rater reliability. The parameters that will be radiographically evaluated are the TAD, the position of the chephalomedulary nail in the femur head and the alignment [42]. Moreover, throughout the follow-up the varus malalignment of the implant will be assessed, as well as the cut-out of the nail and the percentage of nonunion or malunion. Varus collapse is a rotation of the proximal part of the femur anteriorly for at least 3°.

#### Outcome assessment

- Patient reported general health status prior to surgery and at 6-, 12- and 24-weeks post-surgery using the SF-36 form, the EQ-5D-3L Questionnaire, the SARC-F Index and the Elderly Mobility Scale (EMS).
- Pain level at the perioperative period and at 6, 12 and 24 weeks postoperatively using the visual analogue scale (VAS) Pain Score.
- Comparison of perioperative and intraoperative surgical data (delay for surgery, age-adjusted Carlson Comorbidity Index, operation time, fluoroscopy time and dosage, blood loss, length of hospital stay, prescription of pain-killers, osteoporosis assessment using postoperative DEXA of the unaffected hip, union time and intraoperative surgeon related complications, including lag screw malposition, propagation of the fracture, non-anatomical reduction, varus/valgus deformity, rotational deformity and tip-apex distance (TAD) using postoperative CT of the pelvis).
- Patients’ functional status at 24-week follow-up using the patient reported disease-specific scores (Harris Hip score HHS and Oxford hip score-OHS).

#### Follow-up assessments

All the patients should undergo a clinical and radiological follow-up at 6-, 12- and 24 weeks postoperatively. The follow-up will be concluded by antero-posterior radiographs of the pelvis, lateral radiography of the treated hip and questionnaire like HHS, OHS, SF-36, EQ-5D-3L, EMS, Sarc-f, NPRS scale and Vas score.

### Statistical analysis

The primary outcome measure of device radiological failure is considered a binary outcome (device failed/did not fail). A binary logistic regression model will be performed to assess the association between the outcome of device failure and the predictor of device type (Gamma3, Intertan). The complication rate of G3 versus InterTan at 24 weeks post-operatively would be compared using a chi-squared (at the 5% level). The differences between HHS and OHS between groups will be assessed using an independent samples *t* test at 24 weeks postoperatively at the 5% level. Test levels, will be adjusted using the methods of Holm-Bonferroni to allow for the multiple comparisons. A linear regression analysis will also be used to quantify the effects of the treatment groups on each of the primary outcome measures, after adjusting for the effects of a range of other important, potentially confounding, factors (e.g. age, gender) recorded for each patient.

## Discussion

Since the 1960s, the DHS has become the standard implant for surgical treatment of stable intertrochanteric fractures as it allows controlled fracture compression but despite additional modifications, such as trochanteric support plates and antirotational screws, unstable trochanteric fractures are less successfully treated by this method [17–20]. Cephalomedullary nails can provide better lateral wall support in more complex fracture patterns but nevertheless, cut-out of the hip screw has been described as the most frequent mechanical failure for all implants [21–23]. Several studies have shown that the incidence of cut-out for different compression hip screws and cephalomedullary nails ranges from 0 to 16.5% [24–26]. In a recent study by Bojan et al. [27] the primary cut-out rate of Gamma-nail in 3066 consecutive patients was 1.85% and was strongly associated either with unstable fractures involving the trochanteric and cervical regions or the combination of both, non-anatomical reduction and non-optimal screw position which are the only two factors that can be controlled by the surgeon. Recent developments including locking plates, antirotational screws and cement-augmented fixation techniques indicate that the problem of fixation failure is still unresolved [28–31].

The Trigen Intertan (IT) nail (Smith and Nephew) is a current generation IM device, featuring a configuration comprised of a larger superior lag screw and a smaller integrated screw that allows for linear compression of the fragments at the fracture site while providing high rotational stability [32, 33]. Clinical studies evaluating the IT nail against other single screw intramedullary or extramedullary devices have shown controversial results, either similar regarding Harris Hip Score, operation time, blood loss, time to union, mean hospital stay, union problems and perioperative complications [43, 44], inferior, comparing surgical time, blood loss, fluoroscopy usage and intraoperative complications [45–47], or superior in terms of implant failure, mal-union, lag screw cut-out, short term reduction of pain, functional outcomes and uncontrolled varus fracture collapse [33, 48–50].

The aim of this study is to perform a comparative biomechanical testing between a commonly used single screw cephalomedullary nail (Gamma3) and an integrated dual lag screw nail (InterTan) in unstable (AO A2_1–3_) intertrochanteric fractures created on osteoporotic composite femurs. We would hypothesize that the InterTan nail would provide higher stiffness and load to failure and less varus collapse in unstable intertrochanteric fractures after cycling loading. Finite element parametric models of different screw positioning in both implants would be probably reveal the reasons for early or late mechanical complications and implant failures. A prospective, randomized, 2-arm, parallel clinical study will follow including approximately 200 patients. The primary aim would be the differences in radiological measurements (cut-out, varus displacement, loss of reduction, malunion, nonunion) between Gamma3 and IT in closed unstable intertrochanteric fractures (AO A2_1–3_) in patients aged over 70 years old at the 24-week follow-up evaluation. The second primary aim would be the functional status at the 24-week follow-up using patient reported disease-specific scores (HHS and OHS) along with several other perioperative and postoperative data, level of pain and general health status (SF-36, EQ-5D-3L, SARC-F Index and Elderly Mobility Scale).

Recent systematic reviews and metanalyses have reported controversial results regarding the superiority of InterTan to other single or double lag crew cephalomedullary nailing systems. Ma et al. [13] demonstrated that IT was not found to be superior to 1-screw nailing system (Gamma3, PFNA) in terms of Harris Hip Score (HHS), blood loss, total complications, union time, length of hospital stay, revision rate, and fluoroscopy time; in contrast IT showed less implant cut-out rate and femoral fractures when compared with the control groups. The authors concluded that since IT shows similar functional recovery, revision rate and longer surgery time, it is not worthy of being recommended as an alternative intramedullary nail in intertrochanteric fractures.

Nerhera et al. [51] found that InterTan was clinically more effective when compared to a single screw cephalomedullary nail (PFNA) resulting in fewer complications, fewer revisions and fewer patients complaining of pain. No difference has been established regarding non-unions and Harris Hip Score. Intraoperative outcomes favour PFNA with less blood loss and fluoroscopy usage. The authors concluded that further studies are warranted to explore the cost-effectiveness of these and other implants in managing patients with intertrochanteric fractures.

Date et al. [52] showed that PFNA and Gamma3 had better intraoperative outcomes compared with IT; however, IT had superior implant-related outcomes of cut-out and screw migration. No difference was found between IT and PFNA or Gamma3 in Harris Hip Scores, time to union, malunion and nonunion. The authors suggested that further long-term studies are needed to evaluate clinical outcomes and cost–effectiveness of these cephalomedullary devices.

Onggo et al. [53], reported that InterTan was associated with lower complication rates in terms of all-cause revisions, cut-outs, medial or lateral screw as well as persistent hip and thigh pain. In terms of perioperative parameters, InterTan was associated with longer operative and fluoroscopy times. There was no statistically significant difference in terms of clinical Harris Hip Score and radiological outcomes, non-union, haematoma, femoral fractures, varus collapse, length of stay and mean intraoperative blood loss between the 2 groups. The authors concluded that integrated dual lag screw cephalomedullary nails are associated with fewer revisions and complications but there is insufficient data to suggest that either nail construct is associated with better functional outcomes.

Finally, Quartley et al. [54] in their recent metanalysis of 23 studies (17 with unstable fractures) found that IntetTan reduced the risk of revision/reoperation by 64%, implant failures by 62% and hip and thigh pain by 50% in unstable fractures. No differences were noted between IM nail designs for infection rates, healing time, non-union rates, femoral shortening, or Harris Hip Score. The authors concluded that IT nail may reduce incidence of implant-related complications, hip and thigh pain, and the need for revision/reoperation without compromising clinical and functional outcomes.

### Limitations

Our study has several limitations. Firstly, the surgery will be performed by several orthopedic surgeons of our department that have different surgical experience, but they all were trained at the same hospital and the reduction will be closed monitored to be as anatomical as possible. Second, the assessment of fracture healing, cut-out, varus displacement and other complications will be measured with digitalized radiographs that can be misinterpreted by wrong patient positioning and intraobserver variability. Third, there are many factors contributing to fracture healing and regain of functional status that are mostly patient depended, especially in these fragile population of > 70 years old. The main weaknesses of the biomechanical study are its static nature and uniplanar loading configuration. Mechanical failure is a dynamic event in clinical practice, and rotational displacement is equally important to frontal plane displacement postoperatively. However, the current study will test the initial loading characteristics of various lag screw(s) configurations at subcritical stress levels and can serve both as a guide at events at early postoperative ambulation status and as a reference for future dynamic studies with more complex loading characteristics.

## Conclusions

Despite recent advances in the treatment of intertrochanteric fractures, the surgeon related parameters (type of reduction, implant selection and optimal surgical technique) remain the most important factors for a successful clinical and radiological outcome. Taking into consideration the increased health risks related to the treatment of mechanical complications alongside the increased hospitalization and health care costs in the setting of an aging European population, the need to improve treatment outcomes of these fractures is evident. This entails both enhancing our understanding of the prognostic factors of mechanical failure and improving all aspects of intertrochanteric fracture treatment through the optimization of the biomechanical behavior of the fracture-osteosynthesis model by the application of the ideal reduction angle and implant; this is expected to have a positive effect on the rate of mechanical failure and, subsequently, the healing rates, morbidity, and mortality in this fragile patient group.

## Data Availability

The primary investigators are responsible for data storage and availability upon request.
